# Integrating data from different survey types for population monitoring of an endangered species: the case of the Eld’s deer

**DOI:** 10.1038/s41598-019-44075-9

**Published:** 2019-05-23

**Authors:** Diana E. Bowler, Erlend B. Nilsen, Richard Bischof, Robert B. O’Hara, Thin Thin Yu, Tun Oo, Myint Aung, John D. C. Linnell

**Affiliations:** 1 Norwegian Institute for Nature Research – NINA, Box 5685 Torgard, NO-7485, Trondheim, Norway; 2 Faculty of Environmental Sciences and Natural Resource Management, Norwegian University of Life Sciences, Box 5003, NO-1432, Ås, Norway; 3 Department of Mathematical Sciences, Norwegian University of Science and Technology, 7491 Trondheim, Norway; 4 Nature and Wildlife Conservation Division, Ministry of Natural Resources and Environmental Conservation, Nay Pyi Taw, Myanmar; 5 Friends of Wildlife, Room 15, Building 296, Yang-Aung Street, Yankin Township, Yangon, Myanmar

**Keywords:** Conservation biology, Population dynamics

## Abstract

Despite its value for conservation decision-making, we lack information on population abundances for most species. Because establishing large-scale monitoring schemes is rarely feasible, statistical methods that combine multiple data sources are promising approaches to maximize use of available information. We built a Bayesian hierarchical model that combined different survey data of the endangered Eld’s deer in Shwesettaw Wildlife Sanctuary (SWS) in Myanmar and tested our approach in simulation experiments. We combined spatially-restricted line-transect abundance data with more spatially-extensive camera-trap occupancy data to enable estimation of the total deer abundance. The integrated model comprised an ecological model (common to both survey types, based on the equivalence between cloglog-transformed occurrence probability and log-transformed expected abundance) and separate observation models for each survey type. We estimated that the population size of Eld’s deer in SWS is c. 1519 (1061–2114), suggesting it is the world’s largest wild population. The simulations indicated that the potential benefits of combining data include increased precision and better sampling of the spatial variation in the environment, compared to separate analysis of each survey. Our analytical approach, which integrates the strengths of different survey methods, has widespread application for estimating species’ abundances, especially in information-poor regions of the world.

## Introduction

Understanding the patterns in species abundance is essential for the effective conservation of biodiversity. However, for most organisms, we lack standardized monitoring programs at a spatial scale sufficient for conservation and policy needs. Monitoring surveys are usually affected by trade-offs in how effort is distributed^1–3^. Intensive survey methods, such as those collecting species abundance data, often entail a small spatial coverage because of the high effort required for each observation. By contrast, less intensive survey methods, such as those collecting occurrence (presence/absence) data, allow for a larger spatial coverage with the same amount of effort. Hence, a common scenario for a species is that population abundance data are available at specific local sites, while occupancy data are available across a whole region^4^. Methods that integrate the information within datasets on abundance and occurrence to provide more robust and larger-scale population estimates have immediate practical applications^5,6^.

Up until recently, different survey data sets would have been treated in separate analyses, because methods to combine them were not available. However, a growing research area in population and distribution modelling concerns methods that combine different data types in the same analytical framework to take advantage of the strengths in each^4,7,8^. Hierarchical models that separate the ecological processes governing the true dynamics of a system and the observational processes affecting its measurement and recording form much of the basis of this research. There are multiple potential advantages to combining data in a single model for biodiversity monitoring. First, an integrated approach may increase the precision of population estimates because more of the available survey data is used. Second, an integrated approach may increase the spatial scale of inference. This approach has already been shown to be powerful for extrapolating abundance or occupancy estimates to larger geographic scales. For instance, Pagel *et al*.^4^ demonstrated how local abundance surveys could be combined with opportunistic citizen science data to enable region-wide inference on species’ population trends^4^.

Several recent studies have suggested that populations can be modelled as spatial point processes, with spatial points representing individuals within a landscape^6,9^. In this framework, abundance and occurrence surveys are different ways of summarizing the point pattern, and are both dependent on the underlying density of individuals (i.e., the intensity of points in a given area) as well as survey plot size. Hence, both survey types contain information on how the density of individuals varies across the landscape. In fact, it is possible to define a simple mathematical relationship between abundance and occurrence based on occurrence probability being simply the probability of abundance greater than 0^6,10^. While there may be scenarios and scales when occurrence and abundance surveys sample different ecological processes, such as in a metapopulation context, the point process framework is likely to have a broad range of applications to align different types of survey data for statistical integration.

**Figure 1:**
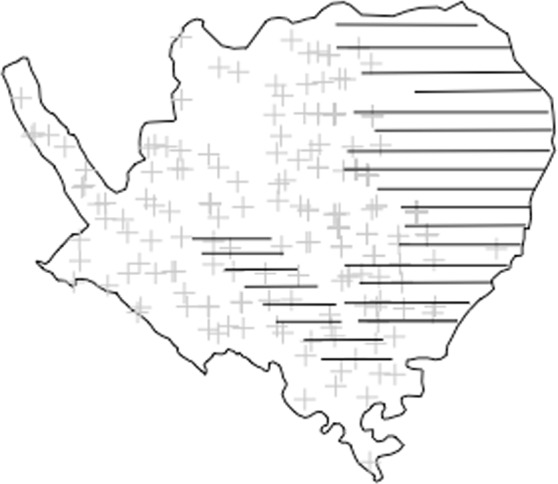
Map showing the distribution of the distance-sampled line-transects (solid horizontal bars) and camera traps (grey crosses) within Shwesettaw Wildlife Sanctuary, Myanmar. We combined the information from both surveys in an integrated hierarchical model. Fig. S1 shows the position of the sanctuary within Myanmar.

Here we integrated data from different survey types using point process theory to estimate the size of a local population of one of the most endangered deer species in the world, the Eld’s deer (*Rucervus eldii*) in central Myanmar. Eld’s deer, also known as the thamin or brow-antlered deer, are native to southeast Asia and live in open and flat forests, especially dry deciduous dipterocarp forests. The species’ range is now highly fragmented and localized within its formerly large range across southeast Asia^11^, due to overhunting and habitat loss^12,13^. Our study population was in the Shwesettaw Wildlife Sanctuary (SWS), one of the two largest remaining populations in Myanmar, which has received much less attention than another population in Chatthin Wildlife Sanctuary. IUCN red list assessments only refer to decades old information from Shwesettaw^14^, and as a result most research and conservation effort has been expended on the Chatthin population. In general, there is very little quantitative or systematic data about the status of Eld’s deer and other wildlife in Myanmar, and the country is entirely absent from global conservation indicators such as the Living Planet Index^15^. Distance sampling surveys of Eld’s deer abundance in SWS began in the 1990’s as a routine activity, but none of the data has ever been published. Camera trap surveys of species’ occupancy patterns were conducted in 2014 and 2016 to assess the structure of the local wildlife community, but also captured images of Eld’s deer. We combined Eld’s deer data from these two sources that both provide information on the number of deer groups across the sanctuary but only partially overlap in space (Fig. 1). We show how a hierarchical model can be used to combine these data by specifying separate observations models for each survey type but a common ecological state model for both the occurrence data (from the camera traps) and abundance data (from the distance sampling survey). We show a novel way to link the different survey types based on the mathematical equivalence between cloglog-transformed occurrence probability and log-transformed expected abundance. First, we tested the generality of our approach using simulation experiments. In these, we tested the effect of integrated models and separate models (of each survey type) to model the variation in deer density across sites, specifically by examining the statistical relationship between an environmental covariate and abundance. Second, we built an integrated model to estimate the current population size of Eld’s deer by modelling the variation in deer density across sites with environmental covariates and spatial spline terms. Finally, we compared our findings on deer abundance, and the factors affecting it, with the understanding that would have been gained by analysis of each dataset separately.

## Results

### Simulations

**Figure 2:**
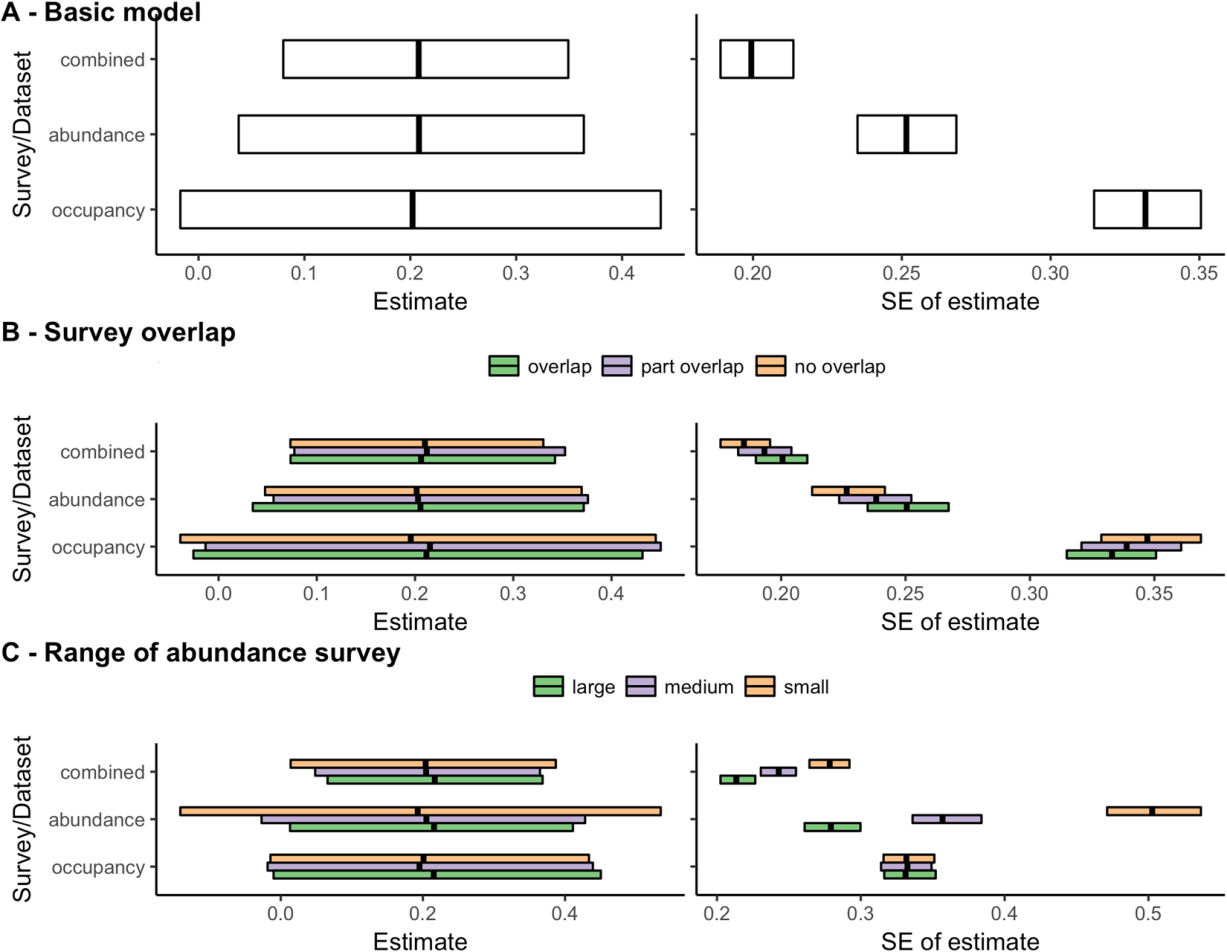
Results of simulations to test the ability to simultaneously model occupancy data and abundance data in an integrated model. Shown are estimates of the effect of an environmental covariate on abundance and the standard error (SE) of the estimated effect based on different models and datasets: binomial model applied to occupancy survey data; Poisson model applied to abundance survey data or a combined model that uses both datasets in a hierarchical model, linking each via a shared ecological process model. Shown are boxplots summarising the results of 1000 random simulations. We test in (**A**) the core model; in (**B**) the effect of survey overlap (overlap = both surveys sampled the same environmental covariates; no overlap = the abundance survey sampled sites with higher covariate values and hence higher abundance while the occupancy survey sampled sites with lower covariate values and hence lower abundance) and in (**C**) the effect of changing the range of the environmental covariate values that was sampled by the abundance survey. The full code for the simulation is provided in the SOM.

We assumed a landscape of 100 sites with varying environmental covariate values that were positively associated with species abundance. Fifty sites were sampled by an abundance survey and 50 sites were sampled by an occupancy survey. In Fig. 2A,we show the main support for our approach: both the binomial model (with cloglog link) applied to the occupancy data and the Poisson model (with log link) applied to the abundance data produce the same estimates of the effect of the environmental covariate on species abundance, and hence the combined model (including both the abundance and occupancy data) does as well. However, the standard error of the estimated effect is much lower in the combined model compared with the other models. This can be explained by the greater sample size and hence information used in the combined model, leading to greater precision of the estimate. These simulations also show that the Poisson model produces a smaller standard error than the binomial model because of the greater information within abundance data compared with occupancy data.

Regardless of the extent of sample overlap, the standard error of the estimated effect of the environmental covariate is lower in the combined model compared with the models for each dataset separately (Fig. 2B). We assumed sample overlap affected the environmental covariate values, and hence the average abundance, sampled by each survey; in the no-overlap scenario, the abundance survey sampled higher abundance sites than the occupancy survey. According to mean-variance relationships of the Poisson and binomial distribution, this affected the standard error estimated in the analysis of each dataset separately; however, in all cases the error is still reduced in the combined model.

The benefit of combining data increases when the abundance survey samples only a small part of the range of environmental covariate values, shown by the reduction in standard error from the Poisson model (of only abundance data) to the combined model (including abundance and occupancy data; Fig. 2C). In further simulations, we found that the reduction in standard error from combining data depends on the detection probability and the number of sites sampled by each survey type. In general, the more individuals detected (either because of a greater number of abundance surveys or a higher detection probability of individuals), the smaller the reduction in standard error by combining data in the same model (Fig. S2).

### Summary of empirical observations

**Figure 3:**
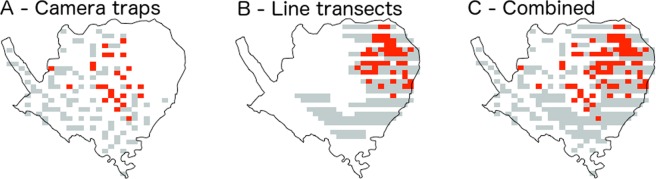
Positive records of Eld’s deer (red squares) out of the total area sampled (grey squares) for the camera trap survey (**A**), line-transect survey (**B**) and their combined coverage (**C**). Each point represents a 1 km square.

Deer were detected in both the line-transect abundance surveys and the camera trap occupancy surveys (Fig. 3). In the line-transect surveys, 25 or 26 deer groups were observed in each survey year, with a mean group size of 7.82 individuals (interquartile range = 3–9.5), leading to total numbers of individuals seen in each year of 167 (2014), 213 (2015) and 214 (2016). These deer groups were distributed across 16 of the 37 3 × 3 km grid cells sampled. During camera trapping, deer group images were captured in 16 of the 49 sampled 3 × 3 km grid cells.

### Ecological processes

Ecological processes were tested at the 3 × 3 km grid scale, which approximated an assumed deer home-range size. The main factor explaining the variation in the number of deer groups across the sanctuary was tree cover; the species was more common in areas with open forest. This effect was detected (i.e., the 95% confidence intervals of the coefficients did not overlap zero) regardless of the dataset analysed (Table 1). However, the effect of forest cover was smaller in the camera trap survey because some deer were observed in the area of higher forest cover. By contrast, deer were not observed in the area of higher forest cover within the sampling area of the line-transect surveys. Both survey datasets also suggested a positive effect of military area; however, the standard error of the effect was larger in the camera trap survey, probably because of the smaller sample size near the military area in these surveys. In the model combining information from both survey types, there were also significant effects of forest cover and military area. Compared to analysis of the line-transect data alone, the combined analysis produced weaker effects of forest cover and a smaller standard error for the effect of the military area (Table 1). There were no effects of field cover in any of the analyses.

**Table 1:** Comparison of the estimated effects of environmental covariates (tree cover, military area and field cover, all standardized to units of standard deviation) on the number of deer groups detected and the estimated total population size (not calculable from the camera trap data) from analysis of each survey dataset.

Data type	Covariate	Estimate	SE	95% CI
Camera data	Tree cover	−1.69	1.97	−4.00, −0.21
	Military area	1.92	1.31	−0.36, 4.56
	Field cover	−0.58	1.29	−2.98, 2.36
	Estimated total population size	—	—	—
Line-transect	Tree cover	−13.11	4.44	−22.98, −5.30
	Military area	0.58	0.24	0.13, 1.07
	Field cover	−0.06	0.28	−0.60, 0.50
	Estimated total population size	1447	242	1028, 1974
Combined	Tree cover	−2.35	0.80	−3.97, −0.76
	Military area	0.49	0.16	0.17, 0.81
	Field cover	0.08	0.23	−0.38, 0.52
	Estimated total population size	1519	270	1061, 2114

Shown is the estimate, its standard error (SE) and 95% confidence interval. For all models, the Bayesian p-values were between 0.4 and 0.5, providing no evidence of a lack of fit.

Only the line-transects provided information on the number of individuals within observed deer groups, i.e., group size. Deer group size was not affected by either habitat (forest cover: 95% CI = −0.08, 0.32; field cover: 95% CI = −0.18, 0.72) or military area (95% CI = −0.30, 0.52). Group size also did not consistently vary among years (95% CI = −0.27, 0.19, 2016 vs 2014).

### Observation processes

In the camera trap survey, the probability to detect a deer group on a given day, conditional on deer using that grid cell, was 8% (95% CI = 6–11%). A detection probability of 8% suggests a 92% probability that deer were detected at least once during a month-long camera trap sampling period. There was an effect of sampling month on detection probability, with a decline over time (95% CI = −0.53, −0.08), and an effect of year (95% CI = 0.04, 1.46, 2016 vs 2014). Detection probability of deer was not related to estimated deer density (number of individuals per km grid) (95% CI = −1.56, 1.15). Also, the effect of a lure at a camera trap was not significant (95% CI = −1.50, 0.47) but the lures were designed to attract carnivores rather than herbivores. The effect of another attractant (water source or salt lick) tended to be positive but did not reach significance (95% CI = −1.0, 18.80). There was no effect of forest cover (95% CI = −1.2, 0.03) on detection probability at camera traps.

Along the line-transects, detection probability of deer groups decreased with increasing distance of deer from the transect line. The effective strip width (ESW) was estimated as 116 m (95% CI = 89, 141). This means that the estimated detection probability (ESW/transect width), which describes the proportion of the line-transect effectively sampled, was 46% (95% CI = 36–56%). Deer group size did not affect the detection-distance distribution (95% CI of sigma of the half-normal distribution = −0.03,0.03), probably because most deer were observed in groups (only 7 of 69 detection events were lone individuals). The detection-distance distribution was also not significantly affected by habitat type (forest cover: 95% CI = −0.05, 0.35), since the 95% confidence intervals overlapped zero.

### Predicted population size

**Figure 4:**
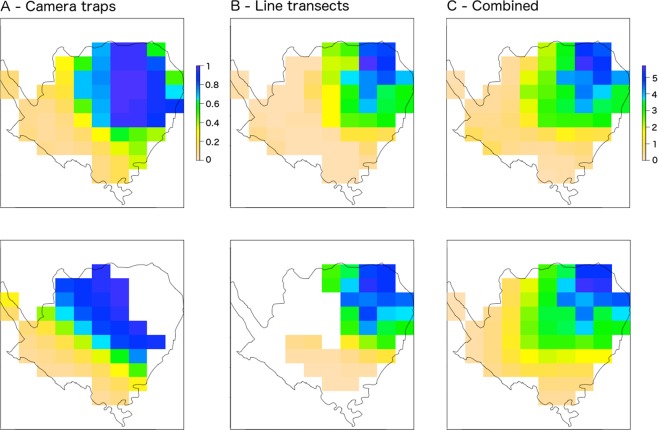
(**A**) Predicted probability of occurrence based only on the camera trapping data; (**B**) predicted density (log number of deer per 3 × 3 km grid cell) based only on the line-transect data and (**C**) predicted density (log number of deer per 3 × 3 km grid cell) based on both datasets. Top row: Predictions were made on the basis of significant environmental covariates (see Table 1) and a spatial spline across the whole study area. Bottom row: Predictions were made using only the smoothing spatial spline model to the area covered by each survey.

Population size was predicted by multiplying the predicted number of deer groups with predicted group size for each sampling grid and summing the values across the whole sanctuary. Overall, the model using the combined data estimated a higher mean deer abundance, by 72 individuals, than the model of the line-transect data alone, although the confidence intervals largely overlapped (Table 1). The higher estimated total abundance can be attributed to the presence of deer, albeit at low density, in the area of higher forest cover within the camera trapping sampling area (Fig. 4), which was not predicted from the line-transect survey data.

## Discussion

Estimates of species population abundance provide essential information for biodiversity research and conservation. Questions about the abundances and trends of species’ populations are ideally resolved using large-scale, well-designed monitoring programs^16^. However, large-scale monitoring schemes have only been achieved for certain taxa (e.g., birds^17^) or have specific methods (e.g., camera traps^18^) designed for specific objectives. Even when local monitoring programs are well-designed for their survey area, they can be difficult to extrapolate to make larger-scale inferences, or apply to questions other than those originally intended^19^. However, quantitative ecologists have begun to develop various methods to combine diverse sources of information from multiple surveys, and even data types, so that reasonable inferences can be made even when the ideal dataset is lacking^4^. Using the example of the Eld’s deer, a threatened species for which reliable estimates of population size are particularly essential, we show how different data types (abundance and occupancy survey data) can be combined in a single hierarchical model to estimate abundance based on all available information across the population.

Only 213 deer were seen in the 2015 line-transect survey; however, this does not equal total population size because of imperfect detection, i.e., only a fraction of individuals present were detected during the survey. The distance-sampling method of the line-transect surveys enabled prediction of the total number of deer in the region sampled by the line-transects; however, the line-transect survey region did not sample the whole sanctuary. By combining data from the line-transects with data from camera trap surveys, we could obtain information on deer detections/non-detections at sites across the entire sanctuary area. The camera trap surveys revealed that the deer occupied areas of the sanctuary with high tree cover, as well as low tree cover, which was not expected based on the information obtained from the smaller geographic range sampled by the line-transect surveys. Hence, we estimated a larger population size in the combined model than in the line-transect only model. However, our analysis confirmed that the line-transect survey covers the majority of the deer population, and hence may be sufficient to estimate population trends of deer over time. We could only reach this conclusion with confidence by testing the effect of integrating the different survey data available.

Our analysis suggests that the Shwesettaw Wildlife Sanctuary is providing, at least over a substantial part of its area, suitable habitat for Eld’s deer and that it likely contains the world’s largest population of the species. Our analysis also confirmed the preference of this species for more open forest habitats, which is consistent with more intensive ecological studies conducted at various scales^20,21^. In addition, the analysis revealed a strong preference for the military area in the northeast part of the study site. Previous studies suggest that Eld’s deer avoid areas of human activity^20,21^. Our findings could reflect positive effects of the opening of the habitat associated with cotton farming in the military area, as well as the reduced human disturbance due to the area having restricted access, and the availability of seasonal water sources associated with irrigation. This result is in line with increasing documentation about the inadvertent positive effect of military areas on wildlife and biodiversity in general^22^. The new robust estimate of the total population size within our study area has major consequences for the conservation of Eld’s deer. Previously, the main focus of international attention has been on Myanmar’s Chatthin population, where the population is reportedly declining^23^. Our results suggest that the Shwesettaw population is the largest wild population, both in Myanmar, and globally.

Hierarchical models allow the integration of multiple sources of information and data because they separate the factors affecting ecological processes from those affecting observation processes^10^. In surveys of species’ populations, it is only the observation process that varies among different survey types – the underlying distribution of individuals, and the ecological factors affecting it, should remain the same in most cases. Hence, we combined different data types with a common ecological process sub-model and separate observation sub-models. Previous approaches to link occurrence and abundance survey data in the same statistical model have been based on the relationship between detection probability and abundance: species are more likely to be detected when their abundance is higher. This relationship can be best estimated when there is spatial overlap between the two survey types^4,24^. Here, we took advantage of the fact that modelling presence-absence data (using the cloglog link) and abundance data (using the log link) is mathematically equivalent^10^. The cloglog link is much under-used in this context given that it helps interpret the results of occupancy binomial regression models, and especially aids comparison with Poisson models fit to abundance data. However, we note that this approach depends on the species being absent from some parts of the landscape – which is a function of both the underlying density of the organism and the plot size at which occupancy is recorded. Our approach also rests on the assumption that the manifestation of a species’ population across the landscape can be described as a spatial point process and that abundance and occupancy surveys are sampling the same ecological processes. This assumption may not be valid in some contexts, such as when the surveys sample at very different spatial grains, or for a metapopulation of independent patches in which patch occupancy and abundance are affected by different sets of processes.

Our simulations suggested that there are multiple advantages to combining data in a single modelling framework. Most importantly, these are (1) a potential increase in precision, and (2) better sampling of the ecological process underlying the point pattern describing the distribution of individuals in the landscape. While we focused our simulations on the estimation of model coefficients rather than abundance directly, these advantages should filter though to abundance estimates since they were based on model coefficients. Increased precision can arise through increasing the amount of data used by the model^8^. Our simulations showed that abundance data contributes more to precision than occupancy data, which is expected since the former contains more information on the underlying point process. However, in our case, the confidence intervals of the population estimates were not narrowed by combining data. This was probably because the camera trap and line-transect data provided different information; primarily regarding whether deer occurred in areas of high forest cover in addition to the more open areas. If both data types are providing similar information, such as about the relationships between population density and environmental covariates, combining different data should improve the precision of the estimates, as shown through our simulations and similar to the findings of other studies^8^. In cases such as ours, when the different datasets do not completely overlap in space, combining different datasets can lead to more extensive sampling of the environmental range of a population, and means that each dataset provides new information about the relationship between an environmental covariate and population density. This was important in our case because the camera trap surveys sampled a larger area of dense forest cover than the line-transect surveys, while the line transect surveys better sampled the military area than the camera trap surveys. In general, sampling over a larger environmental range and including multiple survey methods into one analysis facilitates estimation of the effects of spatially-varying environmental variables.

Neither of our datasets were sampled at random. At a broad scale, camera traps were spread systematically across the sanctuary area, separated by around 1 km. At local scales, they were placed to maximise the probability of capturing medium- to large-sized mammals that were passing nearby. Hence, deer images were probably caught more often than if the camera traps had been placed at random. Our analysis assumed that this upward bias in deer detections similarly affected deer occurrence probability throughout the sanctuary and hence that the variation in occurrence probability among different grid cells remained more or less the same. Only the spatial variation in occurrence probability was used to inform the combined model, and not the intercept of the model that represented the predicted average occurrence. Surprisingly, we did not find that detection probability was related to abundance^4^, which indicates that there may be unmodeled heterogeneity in the data, adding some caution to our results. The line-transects were also not placed at random. Line-transects were concentrated in the part of the sanctuary that was thought to contain the largest proportion of the Eld’s deer population, in the flatter region of open forest. However, there were also several transects in areas of more dense forest cover, making it still possible to test the effect of forest cover within the line-transect survey data.

Although we made use of occupancy data to model the variation in deer density, some amount of high-quality abundance data was essential to predict absolute deer abundance. Our estimation of abundance was rooted in observations from line-transect distance sampling because only the line-transect surveys provided information on deer group size as well as average deer abundance (i.e., the model intercept). An open question is how much high-quality data is necessary for drawing inferences about abundances. In a theoretical simulation study, Dorazio^25^ used information from planned surveys to account for the geographic bias in opportunistic presence data and found that only a small amount of planned data (covering less than 10% of the area) was needed. Further research is needed to derive general results relating to this question.

Population abundance is one of the 22 Essential Biodiversity Variables proposed by the Group on Earth Observations Biodiversity Observation Network (GEO BON) that should be measured to document and study biodiversity change^26,27^. Although there have been calls for large-scale standardized monitoring programs^28^, databases of biodiversity abundance data, such as the Living Planet Index, mostly hold small-scale local datasets on the occurrences and/or abundances of various taxa^29^. However, the availability and accessibility of other types of biodiversity data is rapidly growing, such as through the Global Biodiversity Information Facility^30^ and similar platforms. Although our analysis yielded a rather small-scale population abundance estimate, our approach could also be tested at larger-scales to integrate different types of related data in a single modelling framework.

Our approach could be also applied to other types of survey data providing information on abundance and occupancy data, for instance, abundance indices and presence data (e.g., from citizen science), to ask questions about the effect of environmental covariates on species’ populations or distributions, or population trends over time^4^. However, statistical models can only go so far – a reasonable understanding of the likely observation and ecological processes is necessary to inform the model, as well as well-documented primary data, for the estimation of species’ population sizes and trends. Moreover, the ability to integrate multiple sources of information does not reduce the investigator’s responsibility for ensuring proper study design^25^. However, as data are being increasingly shared and made accessible, integrating information from multiple data sources is likely to be essential for obtaining estimates of species’ population abundances at large scales.

## Methods

### General approach

To integrate the information from both types of survey data, we took advantage of the properties of the cloglog function, $f(x)=\,\mathrm{log}(\,-\,\mathrm{log}(1-x))$. By using this function as the link function in a binomial glm, we modelled occurrence data as the intensity of an underlying Poisson process^10^, making it statistically equivalent to modelling log-transformed abundance. Occurrence *φ* is the probability that abundance is not zero and hence relates to abundance (mean *λ* of a Poisson process) by the following equation:

$$\phi =1-\exp (\,-\,\lambda )$$

which can be re-arranged as:

$$-\mathrm{log}(1-\phi )=\lambda $$

On taking logs, it becomes apparent that *φ* with a cloglog link is equal to *λ* with a log link:

$$\mathrm{log}(\,-\,\mathrm{log}(1-\phi ))=\,\mathrm{log}(\lambda )$$

**Figure 5:**
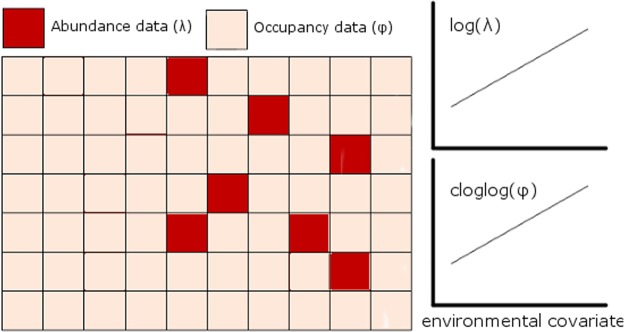
A landscape in which different areas are sampled with different survey methods – one method (e.g. distance sampling – red squares) allows estimation of population abundance and the other method (e.g. camera traps – orange squares) provides information on species occupancy (occurrence or presence/absence survey). Because the effects of an environmental covariate on the intensity of the underlying Poisson process determining population density will similarly affect occupancy and abundance within each grid cell, the same linear model can be fit to each data type with the appropriate link functions (log link for the abundance data and cloglog link for the occupancy data). Hence, both datasets can be used to inform on the slope of the relationship between an environmental covariate and intensity/density.

Hence, by using the cloglog link when modelling occupancy data, as well as a log link when modelling abundance data, the ecological process of different data types can be modelled by the same linear predictor (i.e., the coefficients for the set of environmental covariates) (Fig. 5). At the same time, if included as part of a hierarchical model, separate observation submodels can be used to account for the differences in the sampling process of each survey type. Hence, our approach to combining different data types is to use a hierarchical model to fit a common ecological process model and separate observation submodels that are tailored to account for the specific sampling methods of each survey type.

### Simulations

Before analysis of our real-world population data, we tested our general approach in a series of hypothetical scenarios. We assumed a landscape of 100 sites in which an environmental covariate randomly varied among sites (drawn from a uniform distribution between −1 and 1). Average site abundance across the landscape was set at 10 individuals but expected abundances at each site was positively associated with the site-specific environmental covariate value (effect size = 0.2 log change in abundance per unit change in covariate). Realized abundance at each site was then a random draw from a Poisson distribution with a mean representing site-specific expected abundance. We assumed imperfect detection by the surveyor, based on binomial random sampling, in which p is the detection probability of an individual (base p = 0.1 but see below). In half of the sites (i.e., 50), the number of individuals detected were recorded (abundance survey) while in the other half, only the presence or absence of any individuals was recorded (occupancy survey). We then tested the relationship between the covariate and survey data, by analysing each survey type separately (binomial GLM for the occupancy survey with cloglog link; poisson GLM for the abundance survey with log link) and jointly (allowing the GLMs to share the same regression slope for the effect of the covariate, but each model had its own intercept), and then extracted the slope coefficient for the environmental covariate.

We tested (a) whether we could retrieve the same estimate of the effect of the environmental covariate using the different approaches and effects on its standard error. In (b), we tested the effects of the spatial overlap of the surveys, by varying the values of the covariate over which each survey sampled the data. Under non-overlap, the abundance survey was assumed to sample sites with higher covariate values (from a uniform distribution between 0 and 2) and hence higher abundance, than the occupancy survey (covariate values from a uniform distribution between −2 and 0). In (c), we examined the effects of the environmental range sampled by each survey type, by varying the range of the covariate values over which the survey sampled the data. The abundance survey was assumed to survey a smaller range of values (small range = 50% reduction in covariate value range, uniform distribution between −0.5 and 0.5). We also tested the effects of (d) changing the number of sites at which each survey type was conducted and (e) differences in detection probability of each survey type (results in SOM). Each simulation was run 1000 times. Code for the simulations are provided in the SOM.

### Study area

The survey was conducted in Shwesettaw Wildlife Sanctuary (SWS), situated in the dry zone of central Myanmar (N20°12′, E94°33′), on the western edge of the Irrawaddy plains and adjacent to the foothills at the base of the eastern slopes of the Arakan Mountain range. A total of 552 km^2^ was originally offered various degrees of protection from 1940 as a continuous network of Reserved Forests and was then awarded Wildlife Sanctuary status in 1985. The main motivation for its original creation was to protect its population of Eld’s deer. The present-day boundaries are mostly unchanged apart from the loss of a large part of the northeastern corner to form a military cotton plantation, and several areas along the eastern border that have been lost to agriculture. Most of the area is flat apart from the western edge that forms a series of northwest-southeast aligned ridges rising to 435 m above sea-level. The climate is typical of the dry zone in central Myanmar, with low annual rainfall (average for 2002–2015 = 78 cm), almost all falling between May and October. Temperatures peak in the late dry season (March–April), but over 9 months of the year daily maximums can be in the high 30 s to mid-40 s.

The habitat is mostly forested. Most (c. 80%) of the flat central and eastern part of the sanctuary is covered by a dry upper deciduous forest referred to as Indaing forest in Myanmar. This is characterized by an open structure with grass dominated understory^11^. Key species include *Dipterocarpus* spp and *Shorea* spp., both dipterocarp species. Most of the remaining hilly, western part of the sanctuary is covered with mixed deciduous forest, which contains communities of *Tectona* spp and *Xylia* spp., with a variable understory of bamboos. There are also some areas of dry forest (locally referred to as Than-dhat forest) with *Tectona* spp. as a common community (for general descriptions of this habitat type see)^20,31,32^. There are no human settlements within SWS, but it is tightly surrounded by 39 villages containing 26.000 residents. The landscapes to the east and north are very heavily modified (mainly rice paddy). The areas to the south and west consist of forest-farmland mosaics, but with very heavy human impacts on the remaining forests. One asphalt road bisects the sanctuary from north to south, approximately at the transition between Indaing forest and upper mixed deciduous forest. In addition, the sanctuary is criss-crossed by a dense network of informal tracks and paths passable with motorbikes, bullock carts, or by foot. The sanctuary has always been used by local residents for grazing and obtaining poles, firewood and bamboo. Cultivation was never formally permitted, but clearance of land and seasonal settlement has been ongoing for many decades. This is most significant along the river/stream valleys. There is currently extensive human use of the landscape during the dry season, and illegal killing of wildlife, mainly using snares, occurs.

### Camera trapping

Camera traps were deployed in the dry seasons of 2014 and 2016 between December and April. Traps were systematically placed in a regular arrangement throughout the sanctuary, at least 1 km apart to maximise spatial coverage over the whole area – but the traps could not be placed within the military area boundary. Each microsite for camera placement was chosen to maximise the probability of observing any medium- to large-sized mammals nearby (such as along a trail). Information on potential attractors, such as water sources or salt licks, for the mammalian community were noted. In a few cases, such as when these attractors were absent, a lure (such as fish oil) was placed to act as an attractor for some non-ungulate wildlife but this was not expected to affect deer. The cameras were Reconyx PC 800 Hyperfire Professional Semi-Covert IR, set to take pictures either continuously or in bursts of 3 images with 1-minute intervals if placed near an attractant. Cameras reacted to a combination of movement and thermal difference, used an infra-red flash, and were active day and night. In total, 30 cameras were operated for c. 15 days per trap-site in 2014 and 50 cameras for c. 30 days per trap-site in 2016.

### Line-transect surveys

Distance sampling uses information on the perpendicular distances of observed groups from the transect line to estimate the detection probability and in turn to predict density^33^. We used distance sampling survey data of Eld’s deer collected along line-transects for 2014, 2015 and 2016. A total of 24 line-transects (average length: 8 km; range: 4–13 km) were placed in the approximate same location each year, separated from each other by at least 1 km. The transects were systematically arranged across the areas that were accessible and known to contain deer during the dry season. Transects were walked once each year by 4-man teams during late-March/early-April in the dry season, when visibility was highest. For each deer group seen, the team recorded the sighting distance, the angle of the sighting from the transect line, and the number of individuals in the group. Distances were all estimated by pacing. Locations of groups observed were marked by hand on maps, which were then digitized and georeferenced in decimal degrees. Based on the distance from the observer and the sighting angle, perpendicular distance from the transect line was estimated based on simple trigonometry^33^.

There was only partial overlap between the area covered by camera trapping and by the transects because it was not possible to place camera traps in the military area, and distance sampling was never conducted in the western areas where Eld’s deer had rarely been observed in the dry season (Fig. 1).

### Data harmonization

All data were aggregated onto a 3 × 3-km square grid that approximated the expected home range size of the deer^34^. Eld’s deer in the sanctuary constitute an isolated local population, and hence have little net movement into or out of it. While there may be movements within the sanctuary, this is likely to be seasonal and all our data was sampled during a single season (the dry season). We therefore assumed that the spatial pattern of deer did not greatly change during the sampling period. Observations from the transects were disaggregated to this grid using the spatial coordinates of the groups that were observed. For each grid cell, we calculated the transect length that overlapped with it. This disaggregation was a pragmatic solution to combine the data since only part of it was arranged into transects, and all deer observation along the transects were in any case georeferenced enabling the transfer of observations into grids. The camera trap data were simultaneously modelled at 2-scales (3 × 3 km and 1 × 1 km) but the main ecological factors were tested at the scale of the 3 × 3 km square grid.

### Environmental covariates

**Figure 6:**
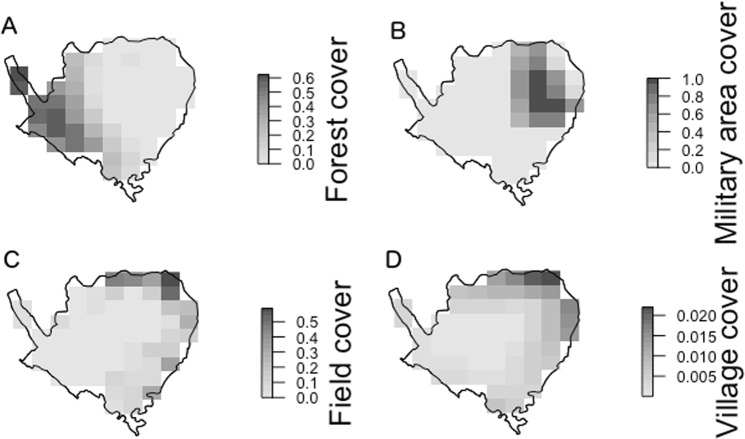
Variation in habitat across the sanctuary: forest cover proportion (**A**) military area cover proportion (**B**) field cover (**C**) and village cover (**D**, average cover of neighbouring cells).

Tree cover data based on satellite images was extracted from the high resolution maps of Hansen *et al*.^35^ available for the year 2000. These tree cover data were used to characterise the variation in the openness of the forest within SWS. Areas of cultivated fields (within the sanctuary) and villages (surrounding the sanctuary) were screen digitized as polygons from visual examination of Google Earth images (Fig. S3) and field visits. For each grid cell, we calculated the proportional area (between 0 and 1) of forest and fields, and village cover (since all villages were outside the sanctuary, village cover was based on the average of a 15 cell × cell neighbourhood matrix). We also determined the extent of overlap with the military area (Fig. 6), hypothesized to be important by the local rangers. All variables were square-root transformed to reduce skew and centred on their means and scaled to units of standard deviation prior to analysis. Since field cover and village neighbourhood cover were strongly correlated (r = 0.8), we focused on field cover since it directly overlapped with the deer distribution, unlike village cover.

### Statistical analysis

To compare the model predictions, we first modelled each dataset separately and then integrated them through a combined model.

#### Camera traps

Using the Eld’s deer images, we created histories of daily occupancy for each camera trap-site during the period when a camera trap was present and functional. Each trap day was considered as an independent repeat survey of deer occurrence during the season, following others^7,36^. Occupancy models were fitted to the data to estimate occurrence probability of deer accounting for imperfect detection^37^. These models account for the fact that absence of deer images at a trap may simply be non-detection rather than true absence. We used a multi-scale model so that we could separately model different factors that vary at different spatial scales^10,37,38^. We tested the main ecological factors affecting occupancy, at the scale of the 3 × 3 km grid, which was assumed to be constant during the study period. However, there were usually multiple camera traps within each 3 × 3 km grid. Hence, we also tested for the effects of smaller-scale environmental variables (i.e., attractor, lure and local habitat) that varied within each 3 × 3 km grid, affecting use of each 1 × 1 km grid at which each camera trap was placed. To this aim, the occupancy data was formatted as a three-dimensional array (dimensions: 3 km grid (*i*), 1 km grid (*j*), sampling day (*k*)). For further information on fitting multi-scale occupancy models, we refer the reader to^10,37,38^.

In our multi-scale occupancy model, we modelled the occurrence state ($z$) as the occurrence probability (*ψ*) per 3 × 3 km grid (*i*) during the survey period, on which we tested the effects of the environmental covariates (forest, military and field cover). We also included a spline term to account for spatial autocorrelation based on the centroid coordinates of each grid cell (*x, y*). The number of knots of the spline was first assessed in a gam using the R package mgcv^39^ by determining the minimum number that resulted in no significant spline term in the model residuals. To develop the BUGS code for the spline, we used the *Jagam* function of the R package mgcv^39^.

$$Occurrence({z}_{i})\sim Bernoulli({\psi }_{i})$$

1 $$cloglog({\psi }_{i})={b}_{0}+{b}_{F}Fores{t}_{i}+{b}_{M}Militar{y}_{i}+{b}_{Fi}Fiel{d}_{i}+s(x,y)$$

We modelled the use (*u*) as the probability of use (*θ*) of a 1 × 1 km grid (*j*) during the survey period conditional on deer occupancy of the surrounding 3 × 3 km grid. We used this level of the model to account for covariates that varied over small spatial scales, i.e., among camera traps in the same 3 × 3 km grid, and rather predicts space use^10^. Specially, we tested whether the probability depended on the presence of a lure or local attractor (a water source or salt lick, grouped since the latter were rare).

$$Use\,({u}_{i,j}|{z}_{i})\sim Bernoulli({z}_{i}.{\theta }_{i,j})$$

$$cloglog\,({\theta }_{i,j})\,=\,{b}_{0}+\,{b}_{A}\,Attracto{r}_{i,j}+\,{b}_{L}\,Lur{e}_{i,j}$$

Finally, we modelled the observed data (y) as the probability of detecting a deer group (*p*) on a given sampling day (*k*) conditional on the use of a 1 × 1 km grid. The probability of detection was allowed to vary with month (as a continuous variable, 0 = Dec; 1–4 = Jan to April), year (reference year: 2016), and habitat (forest cover). In the combined model, we also tested whether detection probability depended on expected deer density, following other studies^4^.

$$Detection\,({y}_{i,j,k}|{u}_{i,j}) \sim \,Bernoulli\,({u}_{i,j}.\,{p}_{i,j,k})$$

$$cloglog({p}_{i,j,k})={b}_{0}+{b}_{M}Mont{h}_{i,j,k}+{b}_{Y}Yea{r}_{i,j,k}+{b}_{D}\,{\rm{l}}{\rm{o}}{\rm{g}}\,Densit{y}_{i}+{b}_{DF}Fores{t}_{i,j}$$

#### Line-transects

We used line-transect data from the most recent years (2014–2016) to estimate current population size, to match the time-period when we also had access to camera trap data. Observations were truncated to those within 250 m from the line-transect to prevent rare and extreme detection distances from biasing the results. As per standard practise, detection probability was assumed to be one for deer groups directly on the transect line^33^. Because individuals within a group are not independent, a detection event was for a group rather than an individual, which matched the definition of a detection event in the camera trap survey. We modelled the observed distances on a continuous scale, but similar results were obtained if the distances were binned into six discrete categories.

The decline in detection probability with increasing distance from the transect line was assumed to follow a half-normal distribution for which the sigma (σ) of the half-normal distribution (more commonly known as the standard deviation, reflecting the spread of the distribution) was estimated in the model^33^. We included a random effect on transect grouping to account for any observer/weather effects on grids that were surveyed as part of the same transect survey. We also tested whether σ was affected by group size or habitat (forest cover) of each detection event *d*.

$${\rm{l}}{\rm{o}}{\rm{g}}({\sigma }_{d})\sim {b}_{0}+Transec{t}_{d}+{b}_{G}GroupSiz{e}_{d}+{b}_{F}Fores{t}_{j}$$

The effective strip width (ESW) of each grid cell *i*, in each year *t*, could be estimated from the sigma of the half-normal detection-distance distribution^40^ as:

$${ES}{{W}}_{i,t}=\sqrt{\frac{(\pi \ast {{\sigma }_{i,t}}^{2})}{2}}$$

Since ${Density}\,({per}\,{unit}\,{area})=\frac{{Number}}{{Area}}$, the number of deer groups observed (Obs) per grid cell could be related to density *D* (defined as the number of deer groups per 9 km^2^ grid cell) as:

$$Ob{s}_{i,t} \sim {Poisson}({N}_{i,t})$$

$${N}_{i,t}={D}_{i,t}\times \frac{({L}_{i,t}\times {ES}{{W}}_{i,t}\times 2)}{9}$$

in which *N* is the expected number of deer groups within the survey area and *L* is the transect length that crosses a grid cell. The division by 9 was necessary so that D represented density per the 9 km^2^ area of each grid cell, rather than density per km^2^. We then tested the effects of the environmental covariates, as well as the spatial spline (in the same manner as in the camera trap data analysis), on density via a log-linear model. Year was included as a fixed factor with the final year, 2016, as the reference year. We also included an observation-level term (*Obs*) to account for overdispersion.

2 $${\rm{l}}{\rm{o}}{\rm{g}}({D}_{i,t})={b}_{0}+{b}_{F}Fores{t}_{i}+{b}_{M}Militar{y}_{i}+{b}_{Fi}Field{}_{i}+Yea{r}_{t}+s({x}_{i},{y}_{i})+Ob{s}_{i,t}$$

All analysis so far was based on modelling the number of deer groups. Information collected during the line-transect also comprised group size of each deer group. Using a log-linear model, we also tested the effects of the environmental covariates on group size *G*, including grid cell as a random factor and year as a fixed factor, as in Eq. 2.

$${\rm{l}}{\rm{o}}{\rm{g}}\,({G}_{i,t})={b}_{0}+{b}_{F}Fores{t}_{i}+{b}_{M}Militar{y}_{i}+{b}_{V}Field{}_{i}+Yea{r}_{t}+Gri{d}_{i}$$

To produce a total population size (*P*), we multiplied the predicted number of deer groups per grid cell for each survey year by predicted group size. We used only the environmental covariates whose 95% CI did not overlap zero for these predictions. This was averaged across years and summed across all grid cells to determine the total population size.

$${P}_{i,t}={G}_{i,t}\times {D}_{i,t}$$

$$P=\sum _{1}^{n}{\overline{P}}_{i}$$

#### Combined model

**Figure 7:**
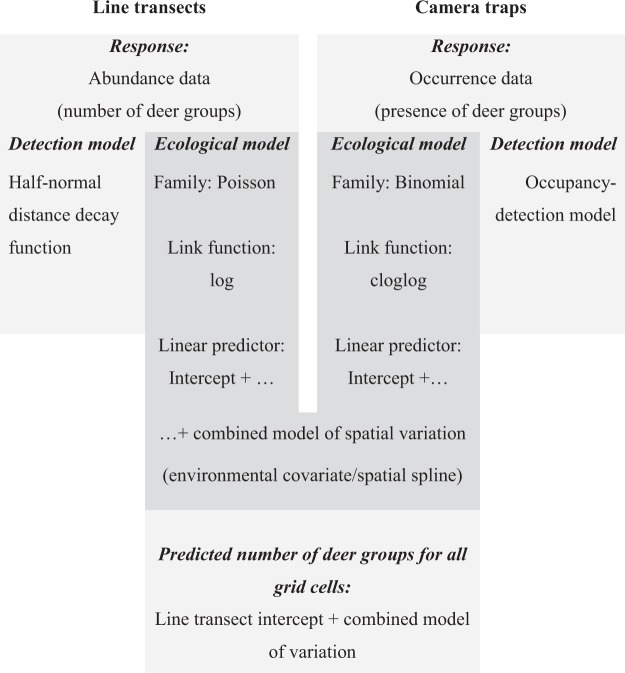
Schema of the combined hierarchical model to demonstrate the links between the abundance model (line-transect data) and the occupancy model (camera trap data).

We repeated most of the steps above except we allowed the survey types to share the same ecological submodel (i.e., Eqs 1 and 2 were linked, Fig. 7). Hence, our model can be regarded as an integrated model since this linear predictor is simultaneously fit in the ecological model using information from both surveys. We did, however, allow the intercept in this ecological model to differ for each data type, to account for the camera-traps providing only relative and not absolute information on deer group density. Since all environmental covariates were mean-centred and standardized to units of standard deviation, the intercepts represent the predictions at mean values of the covariates. Each dataset retained its own observation submodel (Fig. 7).

#### Model fitting

All models were fitted using Bayesian methods with RJAGS using R version 3.4.0. We ran the MCMC chains for 200,000 iterations, discarding the first 50,000 as burn-in. Normal priors with mean = 0 and large variance (100) were used for intercept and slope coefficients. Uniform (0, 10) distributions were used for standard deviations of random effects. However, in the combined model, we used a narrower prior (uniform (3, 6) on the log-scale, based on the estimate from the line-transect data only model) on the estimate of sigma (the parameter of the distance-decay function) since that was primarily informed by the line-transect data. We assessed convergence using the Gelman–Rubin statistic where values < 1.1 indicated convergence. As a posterior predictive check, we calculated a Bayesian p-value in which values towards 0 and 1 indicate a lack of fit^10^. Effects were regarded as significant when the 95% confidence intervals of the model coefficients (reported in the Results section) did not overlap zero. The model code for the simulations and data analysis can be found in the SOM.

## Supplementary information

SOM includes Figs S1 to S3 and code the the simulations and integrated model.

## Data Availability

The datasets generated during and/or analysed during the current study are available via the corresponding author on reasonable request.
