# Supplementary material for: Integrating data from different survey types for population monitoring of an endangered species: the case of the Eld’s deer
Source: Sci Rep. 2019 May 23;9:7766. doi: 10.1038/s41598-019-44075-9 (PMC6533261; doi:10.1038/s41598-019-44075-9)
Supplement: Supplementary file 1 — SOM includes Figs S1 to S3 and code the the simulations and integrated model. [file 41598_2019_44075_MOESM1_ESM.pdf]

Supplementary Information for:

Integrating data from different survey types for population monitoring of an endangered species: the case of the Eld's deer

Diana E. Bowler*, Erlend B. Nilsen, Richard Bischof, Robert B. O'Hara, Thin Thin Yu, Tun Oo, Myint Aung, John D.C. Linnell

Corresponding author: diana.e.bowler@gmail.com

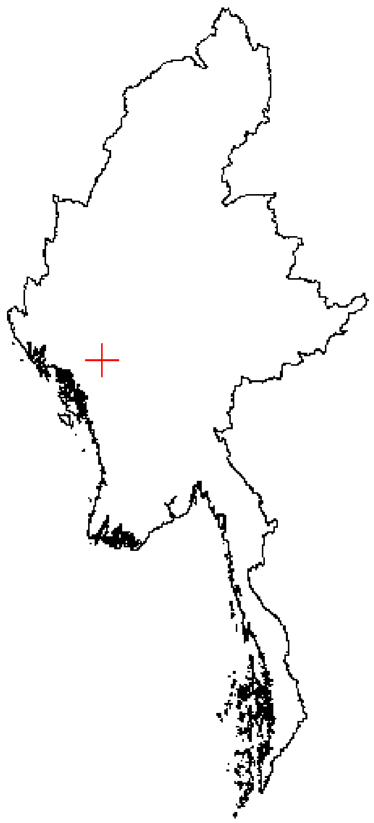

Fig. S1 Location of Shwesettaw Wildlife Sanctuary within Myanmar.

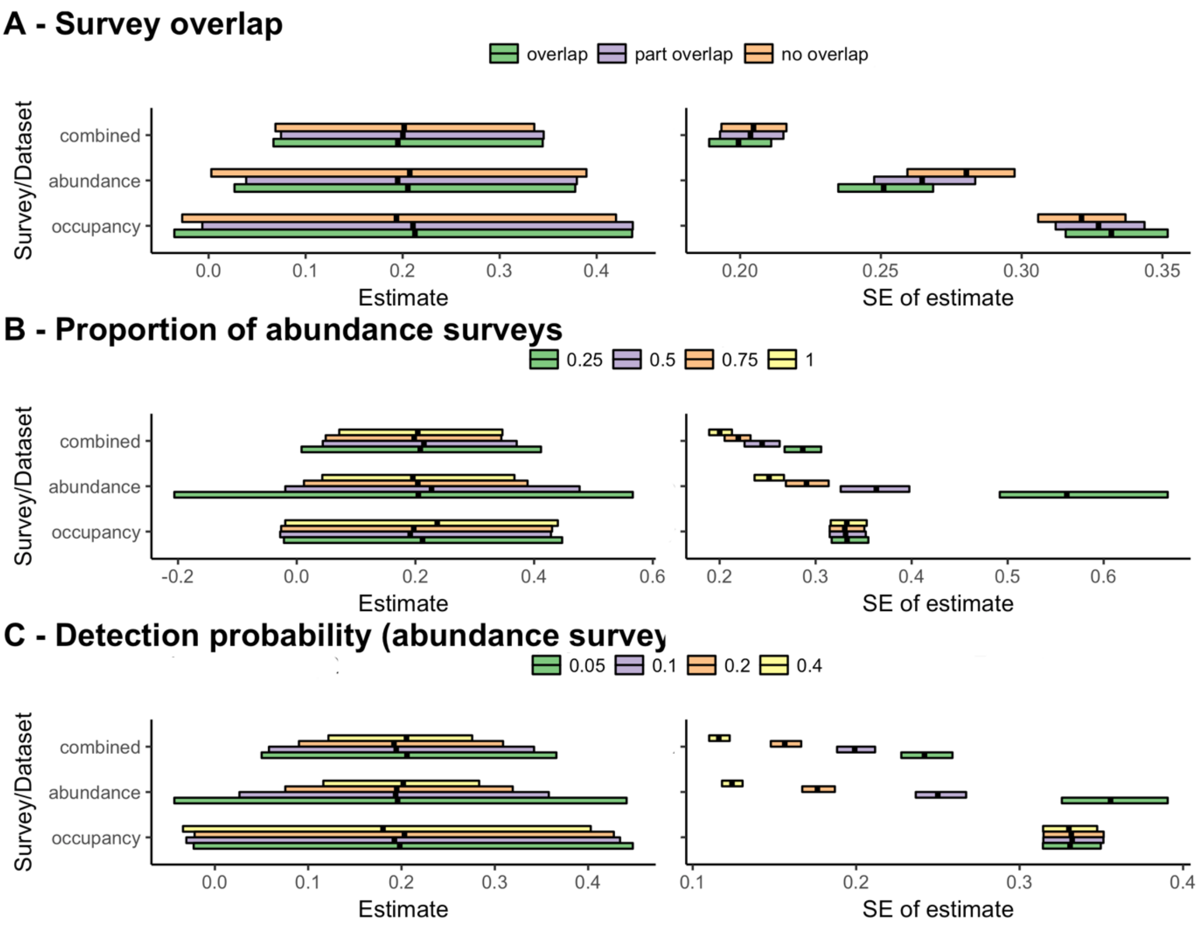

Fig. S2 Results of additional simulations to test the ability to simultaneously model occupancy data and abundance data in an integrated model. Shown are estimates of the effect of an environmental covariate on abundance and the standard error (SE) of the estimated effect based on different models and datasets: binomial model applied to occupancy survey data; Poisson model applied to abundance survey data or a combined model that uses both datasets in a hierarchical model, linking each via a shared ecological process model. Shown are boxplots summarising the results of 1000 random simulations. We test in (A) the effect of survey overlap (overlap = both surveys sampled the same environmental covariates; no overlap = the abundance survey sampled sites with lower covariate values and hence lower abundance while the occupancy survey sampled sites with higher covariate values and hence higher abundance); in (B) the effects of changing the proportion of sites at which there was an abundance survey and in (C) the effect of changing the detection probabilities of individuals during the abundance survey. Full code for the simulation is provided in the SOM.

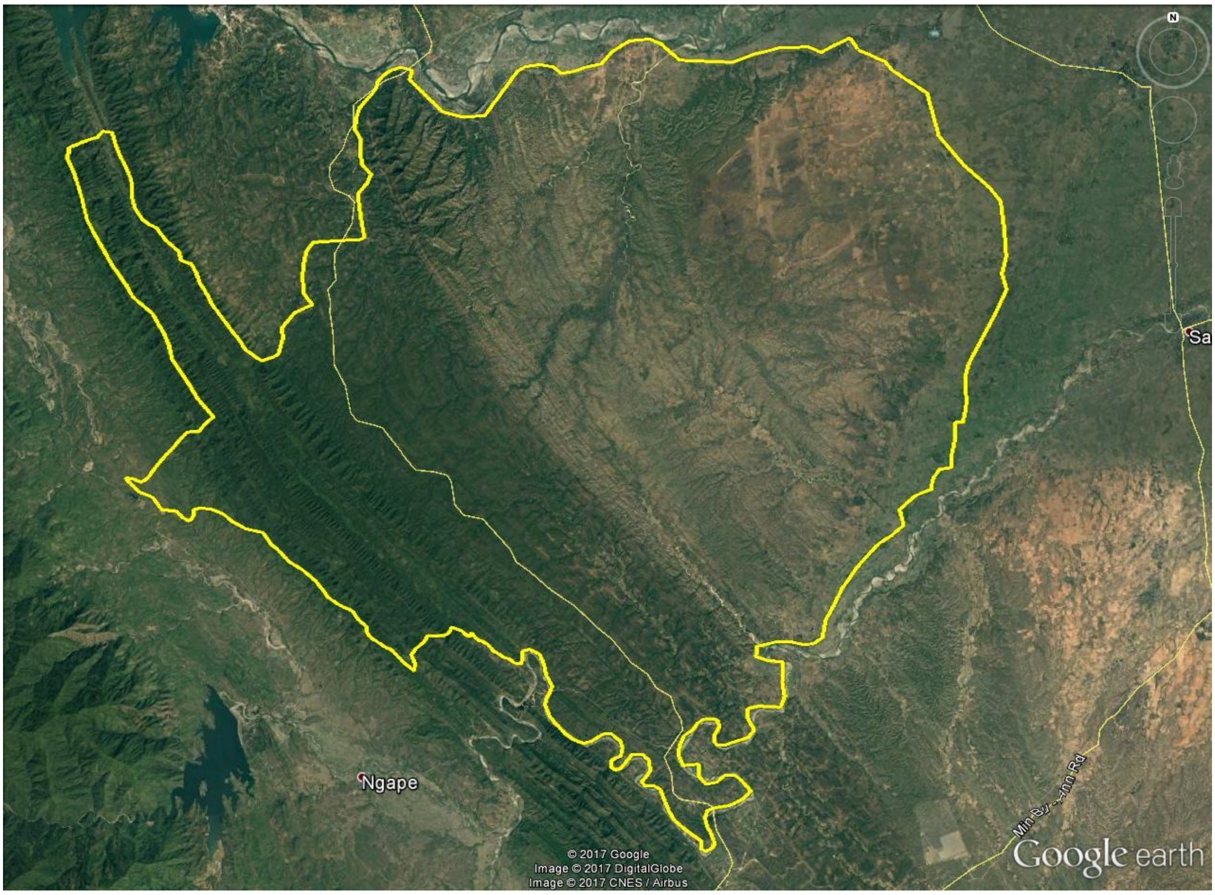

Fig. S3 Google Earth image of Shwesettaw Wildlife Sanctuary, showing the regions of dense forest cover, the road and fields.

```
##################################################################################
#
#Simulations designed to test the equivalence of using the cloglog link on
#presence/absence data and log link on abundance data
#
#Species: We assume the species is distributed in the landscape according to
#a Poisson point pattern. Abundance at each site is affected by a
#spatially-varying environmental covariate (named as forest cover)
#
#Survey: We imperfectly sample the landscapes with two possible survey types:
#survey 1: we record only presence/absence in some sites
#survey 2: we record counts of the number of individuals seen in some sites
#
#Analysis:
#Model 1: Binomial GLM (cloglog link) is fit to the presence/absence data subset
#         with forest cover predictor
#Model 2: Poisson GLM (log link) is fit to the count data subset
#         with forest cover predictor
#Model 3: Combined hierarchical model is fit, combining model 1 and 2 together
#         via a common forest cover predictor
#
##################################################################################

#Specify some basic parameters for the simulated data and parameters that can be
changed:

#total number of sites in the landscape
nuSites<-100

#average abundance of species in a site
abund_int<-log(10)

#effect of the site-specific environmental covariate (fc - forest cover) on
#species' abundance
fc_effect<-0.2

#proportion of sites covered by each survey
survey1_Prop<-1
survey2_Prop<-1

#Detection probability of each survey
detProb_survey1<-0.1
detProb_survey2<-0.1

#range of forest cover sampled bu each survey
survey1_Range<-1
survey2_Range<-1

#sample overlap/range
sampleOverlap="overlap"#overlap perfectly
sampleOverlap="vary_overlap"#abundance survey covers more abundant region
sampleOverlap="change_range"#change survey range of abundance data

#specifies by how much the survey range changes
surveyDifference<-1
```

```
################################################################################

#simulation function

mySim<-function(...){

  #create dataframe
  output<-data.frame()

  for(j in 1:1000){

    #Define a landscape with 100 sites
    sites<-1:nuSites
    dfS<-data.frame(sites)

    #decide which sites are to be surveyed by each survey type:
    sitesH1 <- nuSites*(2/4)
    sitesH2 <- nuSites*(2/4)

    #assume half of sites (i.e., 50) are in the potential study area of each
      #survey type
    dfS$SurveyType<-sample(c(rep(1,sitesH1),rep(2,sitesH2)))

    #Of those in survey 1 area, only collect data at a proportion of them
      #(defined by surveyX_Prop)
    paSites<-round(sitesH1 * survey1_Prop)
    dfS$paCollect<-0#default, no data collection

    #if data to be collected at this site, set to 1 at these sites
    if(paSites<sitesH1){
      dfS$paCollect[dfS$SurveyType==1]<-sample(c(rep(1,paSites),rep(0,(sitesH1-
paSites))))
    }else{#if collecting at all sites
      dfS$paCollect[dfS$SurveyType==1]<-1
    }

    #Of those in survey 2 area, only collect data at a proportion of them
      #(defined by surveyX_Prop)
    abundSites<-round(sitesH2 * survey2_Prop)
    dfS$abundCollect<-0#default, no data collection

    #if data to be collected at this site, set to 1 at these sites
    if(abundSites<sitesH2){
      dfS$abundCollect[dfS$SurveyType==2]<-
sample(c(rep(1,abundSites),rep(0,(sitesH2-abundSites))))
    }else{#if collecting at all sites
      dfS$abundCollect[dfS$SurveyType==2]<-1
    }

    #assign forest cover to these sites
    dfS$fc <- NA

    if(sampleOverlap=="overlap"){

        dfS$fc[dfS$SurveyType==1] <-runif(sum(dfS$SurveyType==1),
                                          -1*survey1_Range,
                                          1*survey1_Range)

        dfS$fc[dfS$SurveyType==2] <- dfS$fc[dfS$SurveyType==1]
```

```
    }else if(sampleOverlap=="vary_overlap"){

      #assume that abundance survey covers region species is more abundant
      #non-overlap but cover same range
      #0 == totally overlap
      #0.5 == 50% overlp
      #1 == 0% overlap

      dfS$fc[dfS$SurveyType==1] <-runif(sum(dfS$SurveyType==1),
                                       -1*survey1_Range-surveyDifference,
                                       1*survey1_Range-surveyDifference)
      dfS$fc[dfS$SurveyType==2] <-runif(sum(dfS$SurveyType==2),
                                       -1*survey2_Range+surveyDifference,
                                       1*survey2_Range+surveyDifference)
    }else if(sampleOverlap=="change_range"){
      #changing survey range of survey 2

      dfS$fc[dfS$SurveyType==1] <-runif(sum(dfS$SurveyType==1),
                                       -1*survey1_Range,
                                       1*survey1_Range)
      dfS$fc[dfS$SurveyType==2] <-runif(sum(dfS$SurveyType==2),
                                       (-1*survey2_Range +
surveyDifference),
                                       (1*survey2_Range -
surveyDifference))
    }

    #generate population abundances:

    #assume there is a linear relationship between forest cover and species
      #abundance

    #assume Poisson error
    abund<-as.numeric()
    for(i in 1:length(sites)){
      log.abund <- abund_int + fc_effect * dfS$fc[i]
      abund[i] <- rpois(1,exp(log.abund))
    }
    dfS$abund <- abund

    #Produce a sampled dataset:

    #For each survey type, each individual has a % chance of being seen:
    dfS$sampleCount_survey1<-
sapply(dfS$abund,function(x)rbinom(1,x,detProb_survey1))
    dfS$sampleCount_survey2<-
sapply(dfS$abund,function(x)rbinom(1,x,detProb_survey2))

    #Survey 1: only record presence/absence data
    dfS$PA<-ifelse(dfS$sampleCount_survey1>0,1,0)
    dfS$PA[dfS$paCollect!=1] <- NA
#set to NA if it wasnt decided to collect data there
    if(sum(dfS$PA,na.rm=T)==(paSites|0)){ next }
#skip iteration if there are no absence sites

    #Survey 2: record the full counts
    dfS$Count<-dfS$sampleCount_survey2
    dfS$Count[dfS$abundCollect!=1] <- NA
#set to NA if it wasnt decided to collect data there
```

```
    #Fit glm to both PA (model 2) and Count data subsets (model 3)

    model1<-
glm(PA~fc,data=dfS,family=binomial(link="cloglog"),na.action=na.omit)
    model2<-glm(Count~fc,data=dfS,family=poisson,na.action=na.omit)#log is
default link function

    #Combine the model coefficients
    output1<-data.frame(names = c("int","slope","int","slope"),
                        model = c("binom","binom","poiss","poiss"),
                        estimate =
c(model1$coefficients[1],model1$coefficients[2],
  model2$coefficients[1],model2$coefficients[2]),
SE = c(summary(model1)$coefficients[1,2],
summary(model1)$coefficients[2,2],
summary(model2)$coefficients[1,2],
summary(model2)$coefficients[2,2]))



    #Model 4: BUGS code for combined model:
    #combined model has common linear predictor but different intercepts
    cat("
        model{

        #common effect of forest cover
        for(i in 1:nsite){
          commonModel[i] <- betaCombined * fc[i]
        }
        betaCombined ~ dnorm(0,0.001)


        #Link to presence/absence data:
        for(i in 1:nsite1){
        PA[i] ~ dbern(psi[i])
          cloglog(psi[i]) <- intPA + commonModel[site1[i]]
        }
        meanPA ~ dunif(0,1)
        intPA <- cloglog(meanPA)

        #Link to count data:
        for(i in 1:nsite2){
        Count[i] ~ dpois(lambda[i])
          log(lambda[i]) <- intCount + commonModel[site2[i]]
        }
        intCount ~ dnorm(0,0.001)


        }
        ",fill=TRUE,file="commonModel_diffIntercept.txt")


    #Compile data for model 3:
    bugs.data <- list(nsite = length(dfS$sites),
                      fc = dfS$fc,
                      nsite1 = length(dfS$PA[dfS$SurveyType==1]),
                      site1 = dfS$sites[dfS$SurveyType==1],
                      PA = dfS$PA[dfS$SurveyType==1],
                      nsite2 = length(dfS$Count[dfS$SurveyType==2]),
```

```
                    site2 = dfS$sites[dfS$SurveyType==2],
                    Count = dfS$Count[dfS$SurveyType==2])

    #Libraries need to run model:
    library(rjags)
    library(jagsUI)

    #run models
    out1 <- jags(bugs.data, inits=NULL, parameters.to.save =
      c("intPA","intCount","betaCombined"),
      "commonModel_diffIntercept.txt", n.thin=5,
       n.chains=3, n.burnin=400,n.iter=1000,parallel = TRUE)


    #Extract summary data
    BUGSoutput<-data.frame(names=c("int","int","slope","slope"),

model=c("BUGScombined_binom","BUGScombined_poiss","BUGScombined_binom","BUGScomb
ined_poiss"),

estimate=c(out1$mean$intPA,out1$mean$intCount,out1$mean$betaCombined,out1$mean$b
etaCombined),

SE=c(out1$sd$intPA,out1$sd$intCount,out1$sd$betaCombined,out1$sd$betaCombined))


output1<-rbind(output1,BUGSoutput)

    #add run details to the output
    output1$simNu<-j
    output1$abund_int<-abund_int
    output1$detProb_survey1<-detProb_survey1
    output1$detProb_survey2<-detProb_survey2
    output1$survey1_Prop<-survey1_Prop
    output1$survey2_Prop<-survey2_Prop
    output1$sampleOverlap<-sampleOverlap
    output1$fc_effect<-fc_effect
    output1$survey1_Range<-survey1_Range
    output1$survey2_Range<-survey2_Range
    output1$nuSites <- nuSites
    output1$nuSites_Survey1 <- sitesH1
    output1$nuSites_Survey2 <- sitesH2
    output1$surveyDifference <- surveyDifference
    output1$PA <- sum(dfS$PA,na.rm=T)

    #add output iteration
    output<-rbind(output,output1)

  }

  return(output)

  }
```

```
################################################################################

#Run model assuming:
library(plyr)

#basic
output=mySim()

#changing overlap of datasets
output <- ldply(c(0,0.5,1),function(x){
  assign("surveyDifference", x, envir = .GlobalEnv)
  mySim()
})

#changing range of survey 2
output <- ldply(c(0.1,0.3,0.5),function(x){
  assign("surveyDifference", x, envir = .GlobalEnv)
  mySim()
})

#increasing amount of abundance data
output <- ldply(c(0.25,0.5,0.75,1),function(x){
  assign("survey2_Prop", x, envir = .GlobalEnv)
  mySim()
})

#change detection probability of survey 2
output <- ldply(c(0.25,0.5,0.1,0.2),function(x){
  assign("detProb_survey2", x, envir = .GlobalEnv)
  mySim()
})

################################################################################
```

```
#JAGS code for integrated model

model {

     #priors for ecological factors
     beta.forest ~ dnorm(0,0.01)
     beta.military ~ dnorm(0,0.01)
     beta.fields ~ dnorm(0,0.01)

     #Common ecological model for both datasets
     for (i in 1:n.grids3km) {#n.grids3km is the number of 3km grids

       #model with spline alone
       #abund.effect[i] <- eta[i]

       #model with covariates and the spline
       #abund.effect[i] <- beta.forest * Forest[i] + beta.military * MilitaryN[i]
       #                 + eta[i] + beta.fields * Fields[i]

       #final model
       abund.effect[i] <- beta.forest * Forest[i] + beta.military * MilitaryN[i]
                         + eta[i]

     }
       #Forest/MilitaryN/Fields are input covariate data

       #Building the spline component
       eta <- X %*% b

       ## prior for s(x,y)
       K1 <- S1[1:4,1:4] * lambda[1]  + S1[1:4,5:8] * lambda[2]
       b[2:5] ~ dmnorm(zero[2:5],K1)

       ## smoothing parameter priors
       for (i in 1:2) {
            lambda[i] ~ dgamma(.05,.005)
            rho[i] <- log(lambda[i])
       }

       ## Intercept
       b[1] ~ dnorm(0,0.01)

       #Different observation models depending on the data:

       #(1) Camera trap data

       #Priors
       theta.lure ~ dnorm(0,0.01)
       theta.water ~ dnorm(0,0.01)
       alpha.month ~ dnorm(0,0.01)
       alpha.year ~ dnorm(0,0.01)
       abund.slope ~ dnorm(0,0.01)
       alpha.forest ~ dnorm(0,0.01)
       alpha.field ~ dnorm(0,0.01)

       #intercepts for each model
       mean.p.ct ~ dunif(0,1)
       int.p <- cloglog(mean.p.ct)
       intercept.ct ~ dunif(0,1)
       int.ct <- cloglog(intercept.ct)
```

```
  int.theta ~ dunif(0,1)
  int.t <- cloglog(int.theta)

#Multi-scale occupancy model
for (i in 1:n.CameraTrapGrids3km) {
    z.ct[i] ~ dbern(psi[i])
    cloglog(psi[i]) <- int.ct + abund.effect[grid.ct[i]]
    #grid.ct specifies which of all grids contain camera trap data
    #abund.effect links to the common ecological model

#use model
for (j in 1:n.1kmGrids){
# Loop over each 1km grid within the 3km grids
    a[i,j] ~ dbern(mu.a[i,j])
    mu.a[i,j] <- z.ct[i] * theta[i,j]
    #full model:
    #cloglog(theta[i,j]) <- int.t + theta.water * Water[i,j] +
                           #theta.lure * Lure[i,j]
    #Water/Lure input data matrices
    #final model:
    cloglog(theta[i,j]) <- int.t

#detection submodel
for (k in 1:n.reps) { # Loop over replicate surveys
    #y.ct is the camera trap presence/absence input data
    y.ct[i,j,k] ~ dbern(mu.ct[i,j,k])
    mu.ct[i,j,k] <- a[i,j] * p.ct[i,j,k]
    #full model:
    #cloglog(p.ct[i,j,k]) <- int.p +
                           #abund.slope * log(Density[site.ct[i]]) +
                           #alpha.year * Year[i,j,k]+
                           #alpha.month * Month[i,j,k] +
                           #alpha.forest * Forest[site.ct[i]]
    #Year/Month/Forest/Fields are input covariate data
    #final model:
    cloglog(p.ct[i,j,k]) <- int.p +
                           alpha.year * Year[i,j,k]+
                           alpha.month * Month[i,j,k]

    }
  }
}

#(2) Line transect data:

# Model for group size
intercept.groupsize ~ dnorm(0,0.01)

#effect of covariates
gs.forest ~ dnorm(0,0.01)
gs.military ~ dnorm(0,0.01)
gs.field ~ dnorm(0,0.01)

#fixed year effect (there are 3 years)
for(i in 1:2){
  yearEffect.gs[i] ~ dnorm(0,0.01)
}
yearEffect.gs[3] <- 0

#random grid effect
```

```
for(i in 1:n.grids3km){
  gridEffect[i] ~ dnorm(0,grid.gs.tau)
}
grid.gs.tau <- pow(grid.gs.sd,-2)
grid.gs.sd ~ dunif(0,10)

for(i in 1:n.Detections){
#n.Detections is number of groups seen along the line transects
  d.Groupsize[i] ~ dpois(exp.GroupSize[i])
  #d.Groupsize is the input data of group sizes of each group detection
  #full model:
  #log(exp.GroupSize[i]) <- intercept.groupsize +
                              #gs.forest * d.Forest[i] +
                              #gs.military * d.Military[i] +
                              #gs.field * d.Field[i] +
                              #gridEffect[dGrids[i]]+
                              #yearEffect.gs[dYear[i]]
  #dGrids indexes the grid of each detection
  #d.Forest/d.Military/d.Field are covariates
  #dYear indexes the year of each detection

  #final model:
  log(exp.GroupSize[i]) <- intercept.groupsize +
                              gridEffect[dGrids[i]]+
                              yearEffect.gs[dYear[i]]
}

#predict group size across all grids
for(j in 1:n.grids3km){
  for(t in 1:n.Yrs){
    grp[j,t] <- exp(intercept.groupsize+yearEffect.gs[t] + gridEffect[j])
  }
}


# Model number of groups
for (j in 1:n.grids3km){
  for (t in 1:n.Yrs){
    #n is input line transect data - it includes NAs where there is no data
    n[j,t] ~ dpois(nHat[j,t])

    #work out area of each cell that is surveyed
    surveyArea[j,t]<-((transectLengths[j,t]/1000)*(ESW[j,t]/1000)*2)/9

    #relate fraction seen to fraction available in whole cell
    nHat[j,t] <- expN[j,t]*surveyArea[j,t]
    log(expN[j,t]) <- abund.effect[j] + yearEffect[t] + obs[j,t]
    #abund.effect links to the common ecological model
  }
}

#fixed year effect prior
for(i in 1:2){
  yearEffect[i] ~ dnorm(0,0.01)
}
yearEffect[3] <- 0

 #random obs
for (j in 1:n.sites){
  for (t in 1:n.Yrs){
```

```
    obs[j,t] ~ dnorm(0,sy.tau)
  }
}
sy.tau <- pow(sy.sd,-2)
sy.sd ~ dunif(0,10)

#predict density (number of individuals per grid cell) for all sites
for(j in 1:n.grids3km){
    for(t in 1:n.Yrs){
      Density.jt[j,t] <- expN[j,t] * grp[j,t]
}
      Density[j] <- mean(Density.jt[j,])
}

#get predicted total across whole area
totDensity <- sum(Density)


#Model for sigma of the detection-distance distribution

pi <- 3.141593

#Priors
b.d.0 ~ dunif(3,6)
b.group.size ~ dnorm(0,0.01)
b.field ~ dnorm(0,0.01)
b.forest ~ dnorm(0,0.01)

#random transect grouping effect
for(i in 1:n.d.transectId){
random.transect[i] ~ dnorm(0,random.transect.tau)
}
random.transect.tau <- pow(random.transect.sd,-2)
random.transect.sd ~ dunif(0,10)

for(i in 1:n.Detections){
 #full model:
#mu.df[i] <- b.d.0 + random.transect[d.transectId[i]] +
            #b.group.size * d.GroupsizeS[i]+
            #b.forest * d.Forest[i]
 #d.Forest/d.GroupsizeS are input covariate data

 #final model:
 mu.df[i] <- b.d.0 + random.transect[d.transectId[i]]

# estimate of sigma
sig.df[i] <- exp(mu.df[i])
sig2.df[i] <- sig.df[i]*sig.df[i]

# effective strip width
esw[i] <- sqrt(pi * sig2.df[i] / 2)
f0[i] <- 1/esw[i]

# estimate sigma using zeros trick
 y[i] ~ dunif(0,W)
 # y are the observed detection distances, W is 250 m
L.f0[i] <- exp(-y[i]*y[i] / (2*sig2.df[i])) * 1/esw[i]
nlogL.f0[i] <-  -log(L.f0[i])
zeros.dist[i] ~ dpois(nlogL.f0[i])
}
```

```
#get average ESW per grid and year
for(k in 1:n.GridYrs){
  for(i in 1:n.Detections){
    grp.ESW[i,k] <- esw[i] * GridYrIdx[i,k]
  }
               ESW.jt[gy.combos[k,1], gy.combos[k,2]] <-
       sum(grp.ESW[,k])/max(1,sum(GridYrIdx[,k]))
}

#predict across all grids
mean.esw <- sqrt(pi * pow(exp(b.d.0),2) / 2)
for(j in 1:n.grids3km){
  for(t in 1:n.Yrs){
    ESW[j,t] <- ifelse(equals(ESW.jt[j,t],0),mean.esw,ESW.jt[j,t])
  }
}


}
```
